# Supplementary figures and images for: Development of models of care coordination for rare conditions: a qualitative study
Source: Orphanet J Rare Dis. 2022 Feb 14;17:49. doi: 10.1186/s13023-022-02190-3 (PMC8843018; doi:10.1186/s13023-022-02190-3)

Appendix 1. A summary of the taxonomy and findings presented in [15]


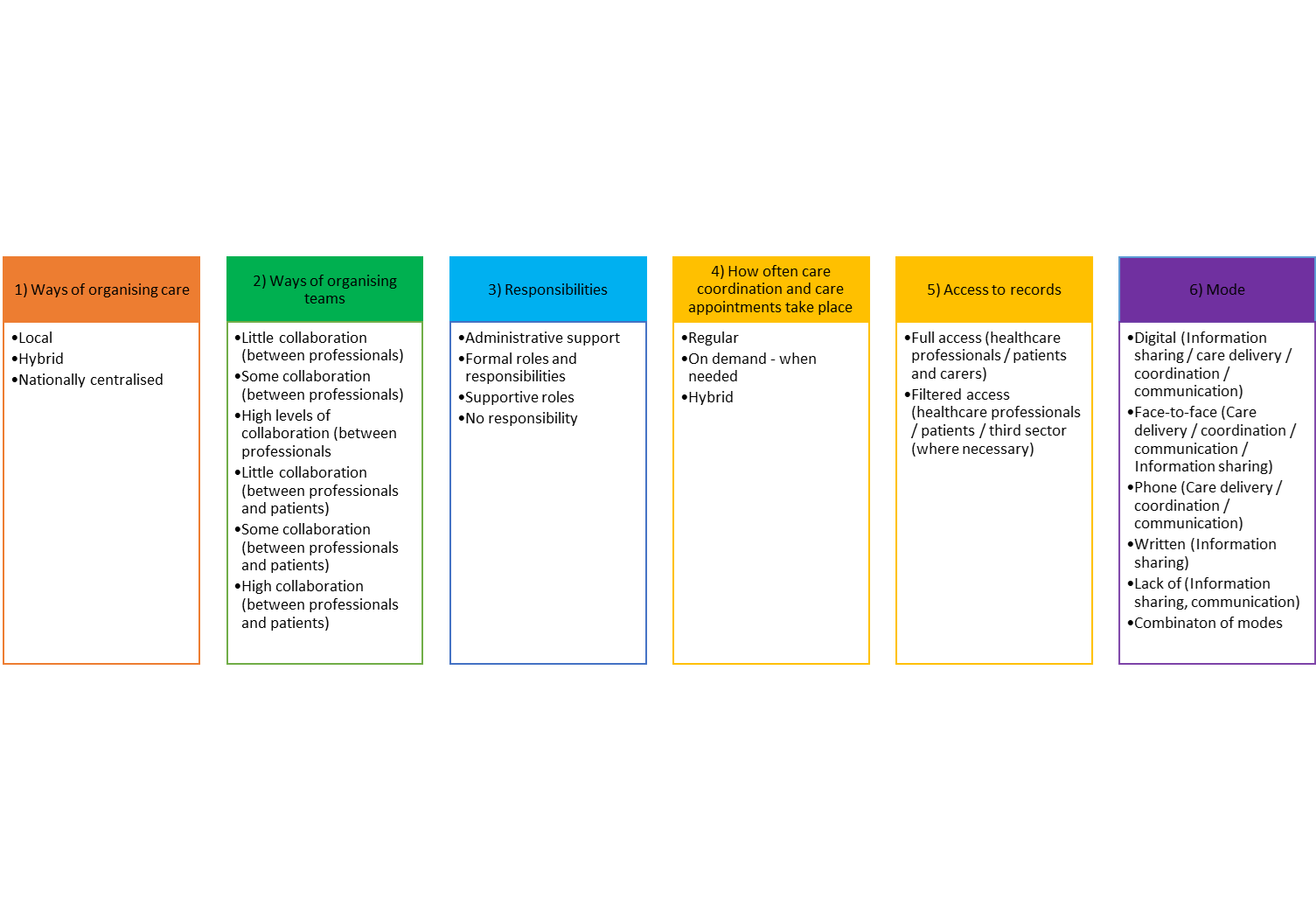

Supplement: Supplementary file 1 — Additional file 1. A summary of the taxonomy and findings presented in [17]. [file 13023_2022_2190_MOESM1_ESM.docx]

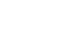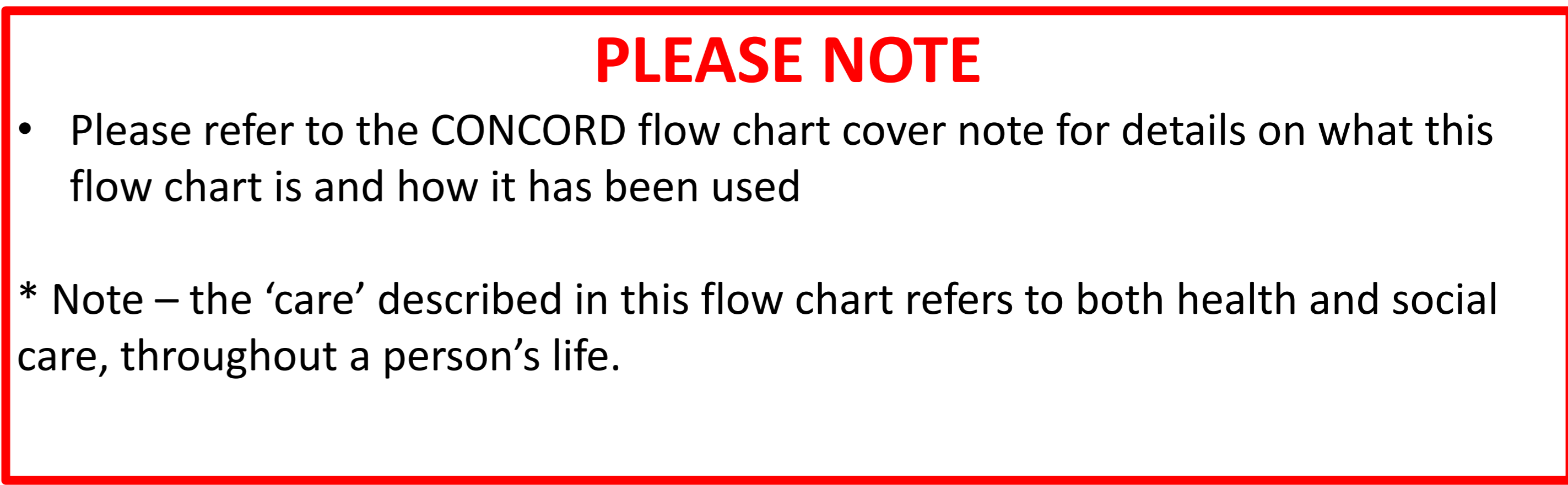

Supplement: Supplementary file 4 — Additional file 4. CONCORD flow chart. [file 13023_2022_2190_MOESM4_ESM.pdf]
