# Supplementary material for: Development of models of care coordination for rare conditions: a qualitative study
Source: Orphanet J Rare Dis. 2022 Feb 14;17:49. doi: 10.1186/s13023-022-02190-3 (PMC8843018; doi:10.1186/s13023-022-02190-3)
Supplement: Supplementary file 2 — Additional file 2. Detailed methods (amended from [9, 17] and Morris et al, forthcoming - CONCORD final report). [file 13023_2022_2190_MOESM2_ESM.docx]

**Appendix 2.** Detailed methods, (amended from [9,15; Morris et al, forthcoming – CONCORD final report])

**Design**

Our study used qualitative methods (interviews, focus groups and workshops).

Using a qualitative approach allows for a more in-depth understanding of complex phenomena (Bogardus et al, 1998; Bradley et al, 2001). Additionally, the use of qualitative methods allows for the direct involvement of those with most experience in the phenomena being studied and classified, such as patients, health care professionals and carers. This is particularly important in health care service research, in which patients, carers and health care professionals are the key stakeholders (Ferris et al, 2018). By understanding patients’, carers’ and health care professionals’ views on the organisation of care coordination for rare diseases we could improve health care services but also optimise the patient experience. It has also been proposed that qualitative studies are well suited to explore new concepts (Bradley, 2001). As coordination of care is a relatively new field, using qualitative methods will offer a rich perspective on care and stakeholders’ preferences.

This research was conducted in a two-stage process. First, interviews (n=30) and focus groups (n=4) were conducted to develop an initial taxonomy. Interviews and focus groups were felt to be appropriate methods for exploring and gathering in-depth perspectives on stakeholders experiences of coordination and the different models of care coordination that currently exist, together with preferences for potential new models of coordination. Workshops (n=2) were then conducted to refine the proposed taxonomy. Workshops were felt to be appropriate for helping to gather consensus around whether the taxonomy was appropriate and to develop recommendations to improve the taxonomy.

**Sample**

We recruited a range of patients with rare, ultra-rare or undiagnosed conditions, carers/parents, health care professionals, charity representatives and commissioners to take part in the interviews (n=30), focus groups (four groups of between six to eight participants) (Kreuger et al, 2002) and workshops (two workshops, one for patients/carers and one for professionals, of approximately 15 participants each). We originally planned for five face-to-face workshops, however we had to reduce this to two remote workshops (due to the COVID-19 pandemic). Interview and focus group participants informed the development of the taxonomy. Workshop participants informed the refinement of the taxonomy.

To take part in the study, participants needed to be aged ≥ 18 years. Children were not included due to ethical issues recruiting participants aged < 18 years. One focus group participant withdrew from the study after the focus group, thus resulting in 22 patients and carers taking part in the focus groups.

Participants were recruited using a range of methods, including email invitation, social media, via the voluntary sector and through our partnerships with four NHS sites.

As there are currently between 6,000 and 8,000 rare diseases (Rare Disease UK, 2018), it was not possible to include participants affected by every rare disease. To ensure that different models of coordinated care (including different types of care coordination and no coordination) and a wide range of experience and expertise were captured, we used purposive sampling. We sampled professionals based on their area of the UK, job role, and experience with different types of care coordination. We sampled patients and carers based on their area of the UK, condition, role, age and experience with different types of care coordination.

### Measures

To gather data to inform the development of the taxonomy, two topic guides (one for interviews and one for focus groups) were developed and used to collect data (see *Supplementary material file 2*). Questions focused on: stakeholders’ experiences of coordinated care; implications of coordinated care; preferences for key aspects of care coordination (including preferred way of coordinating care, format, access, frequency, location, information sharing and transition); benefits and challenges and factors that help and get in the way of coordination. Feedback on the topic guide was sought from the CONCORD PPIAG prior to data collection.

To gather data to refine the taxonomy, one topic guide for both workshops was developed and used to collect data (see Appendix 2). The topic guide was based around the six categories identified in the taxonomy and included prompts regarding whether the participants had feedback on the category (e.g. if we had missed anything and whether findings seemed appropriate based on participant experiences); appropriateness of options in light of the COViD-19 pandemic; and recommendations to improve the category.

### Procedure

Participants were recruited using a range of methods, including email invitation, social media, via the charity sector, and through our partnerships with four NHS sites. Potential participants were asked to contact the study researcher via email or telephone.

To ensure that a range of participants with different experiences were recruited, potential participants were asked to provide responses to eligibility questions when registering their interest. Participants were sent these eligibility questions by email. For professionals, these included: their occupation, speciality and geographical region. For patients or carers, these included whether they receive coordinated care (specialist service and who coordinates), whether they have a diagnosis, age range, ethnicity, geographical region and role. The researcher checked that participants met the eligibility criteria for the study.

Selected individuals were asked to complete consent forms (one for the researcher and one for the participant) prior to taking part in the interviews, focus groups or workshops. Participants who took part virtually or via telephone were asked to return written consent forms in advance. Participants were informed that their data would be kept confidential, fully anonymised and that they could withdraw at any time without providing a reason. Focus group participants were informed that any data collected up until the point of withdrawal would be kept due to difficulties removing individual participants from focus group data. We took steps to ensure that quotes from the participant who withdrew from the study were not included in publications. These steps included removing withdrawn quotes from the analysis spreadsheet.

To gather data to inform the development of a taxonomy, interviews with health care professionals, charity representatives and commissioners, and focus groups with patients and carers, were conducted.

One researcher conducted interviews either by telephone or face-to-face, depending on participants’ preferences. The interviews lasted approximately one hour (range: 44-74 minutes). Two researchers (HW and AS) conducted the four focus groups (one researcher facilitated, and one researcher took notes) (Kreuger, 2002). A third researcher observed one of the focus groups (EH). Two focus groups were conducted face-to-face (one in London, one in Birmingham), and two were conducted virtually using Skype for Business. Focus groups were up to three hours in length (including a break) (range: 149-154 minutes). Interviews and focus groups were digitally recorded using an encrypted dictaphone (with consent from participants) and professionally transcribed. Transcripts were checked for accuracy and fully anonymised (including names and places). Data were stored in the UCL Data Safe Haven (a secure electronic environment, certified to ISO27001 information security standard and conforms to the NHS Information Governance Toolkit) and coded using NVivo 12.

To refine the taxonomy, workshops were conducted virtually. Workshop participants were sent a brief 15-minute video prior to the workshop which outlined the findings of the taxonomy (including each domain and the options within each domain). The presentation also covered qualifier findings including preferences, barriers/facilitators, factors influencing coordination and benefits/challenges of different options. During the workshops, participants were given an introduction to the workshop before being split into three breakout groups. Each breakout group had one facilitator (HW, EH, AIGR) and one note taker (JJ, SM, AH). During the breakout groups, facilitators encouraged the small groups to discuss each of the six domains in the taxonomy. Facilitators prompted about whether participants had feedback on the category (i.e. whether anything had been missed, whether findings seemed appropriate based on participant experiences), appropriateness of options in light of the pandemic and recommendations to improve the category. After the breakout groups, participants reconvened in the main group and each group provided feedback on their discussions. Workshops were recorded using an encrypted dictaphone. Notes were checked for thoroughness, and summarised prior to being sent to a graphic facilitator (New Possibilities, Birmingham, UK) to create a graphical representation of the findings.
